# Impact of COVID-19 on healthcare programs in Zimbabwe: a mixed methods study

**DOI:** 10.1186/s12889-025-22791-4

**Published:** 2025-05-13

**Authors:** Nicholas Midzi, C. Haruzivishe, E. Gonese, S. Sembuche, M. J. Mutsaka-Makuvaza, R. Ayebare, T. Muwonge, S. Nakasendwa, C. Mateta, T. Madanhire, C. N. Chaibva, C. Gwatiringa, K. Mutsaka, I. Phiri, M. Abdulaziz, P. C. Kabwe, V. Dube-Mawerewere, R. Tajudeen, M. P. Fallah, M. Dobbie

**Affiliations:** 1 National Institute of Health Research (Ministry of Health and Childcare), Harare, Zimbabwe; 2 University of Zimbabwe, Harare, Zimbabwe; 3 Africa Centers for Disease Control and Prevention, Addis Ababa, Ethiopia; 4 University of Rwanda, Kigali, Rwanda; 5 Infectious Diseases Institute, Makerere University, Kampala, Uganda; 6 National University of Science and Technology, Bulawayo, Zimbabwe; 7 Epidemiology and Disease Control (Ministry of Health and Child Care), Harare, Zimbabwe; 8 Public Health Division (Ministry of Health and Child Care), Harare, Zimbabwe

**Keywords:** COVID-19, Healthcare service indicators, Healthcare workers, DHIS2, Public health, Health systems impact, Zimbabwe

## Abstract

**Background:**

The COVID-19 pandemic disrupted healthcare services. Understanding similar epidemic-related disruptions on a broader scope in our local setting is critical for the effective planning of essential services. The study determined the impact of Coronavirus disease(COVID-19) on healthcare programs in Zimbabwe.

**Methods:**

A mixed-methods study compared healthcare service delivery trends from the Ministry of Health and Child Care before, during and post the pandemic. It employed two methods of data collection: Key informant interviews (KII) and secondary data analysis from the Zimbabwe District Health Information Systems 2 (DHIS2). Purposive sampling obtained key informants for interviews whilst 18 healthcare service indicators were identified from the national database. Statistical analysis consisted of an interrupted time series analysis of those indicators preceded by visualization to appreciate trend change. An inductive approach was used to code and identify basic themes which were then triangulated against DHIS2 findings.

**Results:**

The study revealed that COVID-19 had a negative impact on health service delivery; increasing disruptions of critical healthcare services, maternal and child health, reproductive health issues, and other specialist services were prominent. The rise in maternal and child mortality cases and caesarean sections could be directly linked to the decline in service delivery during the pandemic. Mitigation strategies that were introduced during the pandemic included the use of community-based services, outreach services, capacity building, and de-congestion of public services.

**Conclusions:**

The pandemic disrupted healthcare delivery, causing service usage to decline due to lockdowns. Response strategies included community services, capacity building, and stakeholder engagement. Future readiness requires epidemic plans, enhanced resources, a multisectoral approach, workforce training, and public education.

**Supplementary Information:**

The online version contains supplementary material available at 10.1186/s12889-025-22791-4.

## Background

The global COVID-19 pandemic, triggered by the Severe Acute Respiratory Syndrome Coronavirus 2 (SARS-CoV-2), has resulted in significant consequences worldwide [1, 2]. As of December 2023, approximately 772 million COVID-19 cases and seven million deaths have been reported globally [3]. With millions of individuals infected and nearly five million lives lost by late October 2021, the impact has been profound and far-reaching [4]. Approximately 12.0 million cases have been reported in Africa, while 254,669 persons have succumbed to COVID-19 in 55 African Union Member States, corresponding to a low case fatality rate of 0.23% [5]. In Zimbabwe, approximately 257 666 cases with 5604 deaths had been recorded as of October 6, 2022 [6].

The COVID-19 pandemic impacted negatively on all sectors either directly or indirectly, and has put an added strain on the already overburdened healthcare system [7–9]. Amidst these challenges, countries are faced with the pressing need for adaptable solutions and collaborative efforts on a global scale [10]. The disruption of services, especially in low-income settings put pressure and stretched most beyond the country’s capabilities [11, 12], whilst revealing prevalent gaps within the preventive and curative health delivery service both for communicable as well as noncommunicable conditions [13, 14].

Studies have shown disruptions across services for all major health areas [9, 15, 16] with the WHO reporting that 90% of the countries experienced disturbances in essential health services including sexual, reproductive, maternal, newborn, child, and adolescent health, immunization, nutrition, mental, neurological, and substance use disorders, HIV, hepatitis, TB, and malaria across all countries regardless of income status [17]. In addition, key informant reports indicated the most disrupted services as outreach (70%), facility based (61%), non-communicable diseases diagnosis and treatment (69%), family planning and contraception (68%), diagnosis and treatment of mental health disorders (61%) and cancer diagnosis and treatment (55%) [17, 18].

Therefore, understanding and documenting similar disruptions on a broader scope in our local setting remained critical for effective planning for the continuity of essential services amidst public health events such as pandemics. To better understand the impact of the pandemic on various services, such as childhood routine immunization and mental health, and several others, it is crucial to identify the extent of disruptions in health service delivery associated with the pandemic between 2020 and 2022 to inform strategic planning for health system recovery [19, 20].

Among other strategies, motivating health workers to vaccinate and to document the potential impact of the COVID-19 vaccine program on healthcare systems in Africa has been well promoted [21, 22]. These efforts require implementation research to inform the national vaccination program to produce the intended outcomes and achieve the vaccination target for the country. Vaccination campaigns as a mitigation strategy in Africa, Zimbabwe included, were however affected by the scarcity of vaccines, such that priority was given to health workers, teachers, and older people [23]. Nonetheless, despite the increased access to efficacious COVID-19 vaccines, many remain unvaccinated due to prevailing misconceptions about the vaccines [24, 25].

Thus, this study assesses the impact of COVID-19 on healthcare programs and generates evidence to inform Zimbabwe’s management of the COVID-19 pandemic, make it more efficient, flexible, and effective, and save lives and livelihoods in the country.

## Methodology

### Study design

This convergent parallel mixed methods study compared healthcare service delivery trends from the Ministry of Health and Child Care (MoHCC) before and during the pandemic from the (i) Key Informant Interviews (KII) and (ii) the Zimbabwe District Health Information Systems 2 (DHIS2).

### Study population

The study included key representatives from the Zimbabwe MoHCC, specifically the national heads of departments and interviewed them as key informants whilst a secondary analysis of the DHIS2 provided quantitative data for analyses. Data collection was performed from 01 June 2023 to 31 August 2023.

### Sampling methods

#### Key informant interviews (KII)

Purposive sampling was conducted to obtain the required key informants for interviews. Representatives (*n* = 15) from the Zimbabwe MoHCC, development and implementing partners for critical programs such as the national expanded program on immunization, primary health care, HIV/TB programs, communicable and non-communicable diseases programs, gender and sexual-based violence programs, blood transfusion services, malaria control, and Antenatal/postnatal services.

### DHIS2

Purposively, key national healthcare service indicators (*n* = 18) were identified from the national database.

### Data collection methods

Key informants responded to guided questions developed for this study on the impact of COVID-19 in their respective institutions, departments, and service areas (Supplementary File 1). The guide covered a range of topics related to the impact of the pandemic on healthcare infrastructure, service delivery and utilization, resource allocation, and response strategies. In addition, the data extracted from the Zimbabwe DHIS2 MoHCC, determined the performance of 18 indicators from 7 health service departments from 1 January 2018 to 31 May 2023 (Table 1).

**Table 1:** Study departments and indicators

Topic/theme	Key Indicators
1. Maternal Health	a) ANC 1st visit, b) Pregnant women completing 4 ANC visits c) Skilled birth attendants d) Institutional deliveries in health facilities e) Caesarian Sections f) Family Planning: new visits and revisits
2. Child Immunisation	a) Pentavalent vaccination 3rd dose b) BCG doses administered < 1 year c) Measles vaccination 1st dose
3. Tuberculosis	a) New cases of tuberculosis diagnosed b) Incident tuberculosis cases among people living with HIV c) Incident tuberculosis cases in children aged 0–14 years d) Number of cases of TB receiving treatment for TB
4. Mortality	a) Fresh stillbirths b) Maternal deaths
5. Nutrition	a) Severe wasting
6. Mental health	a) Mental outpatient visits
7. Malaria	a) New malaria cases

### Data analysis techniques

#### Key informants

Data from the qualitative interviews were analysed using thematic analysis in an inductive manner [26]. Firstly, using three randomly transcribed audio, a data analysis pair developed the initial coding framework by reading line by line of each of the three transcripts to generate the first set of codes. Secondly, the coding framework was then entered into NVIVO 12 Plus for Windows 11 as nodes for analysis. Thirdly, all the transcripts were then imported and coded in NVIVO 12 Plus. An original codebook and coding summary were generated, and the data analysis team developed a narrative, including illustrative quotes, bridging the original research concerns with the participant’s subjective experiences.

### DHIS2

The statistical analysis consisted of an Interrupted time series analysis of those indicators preceded by visualization to appreciate trend change. Model selection was done through AIC minimization. ACF and PACF suggested adequate values for the parameter selection of the parameter p. Model validation was mainly done through residuals check, and most importantly, the values predicted by our model in the absence of COVID-19 (counterfactual) were compared with the observed values.

### Results and findings

The COVID-19 pandemic had a significant impact on the delivery of healthcare services. The use of healthcare and support services declined due to the lockdown and restrictions on movement. When the respondents were asked about their thoughts on how the pandemic affected service delivery, their responses showed that the pandemic significantly impacted access to health services.

### Maternal health

**Fig. 1:**
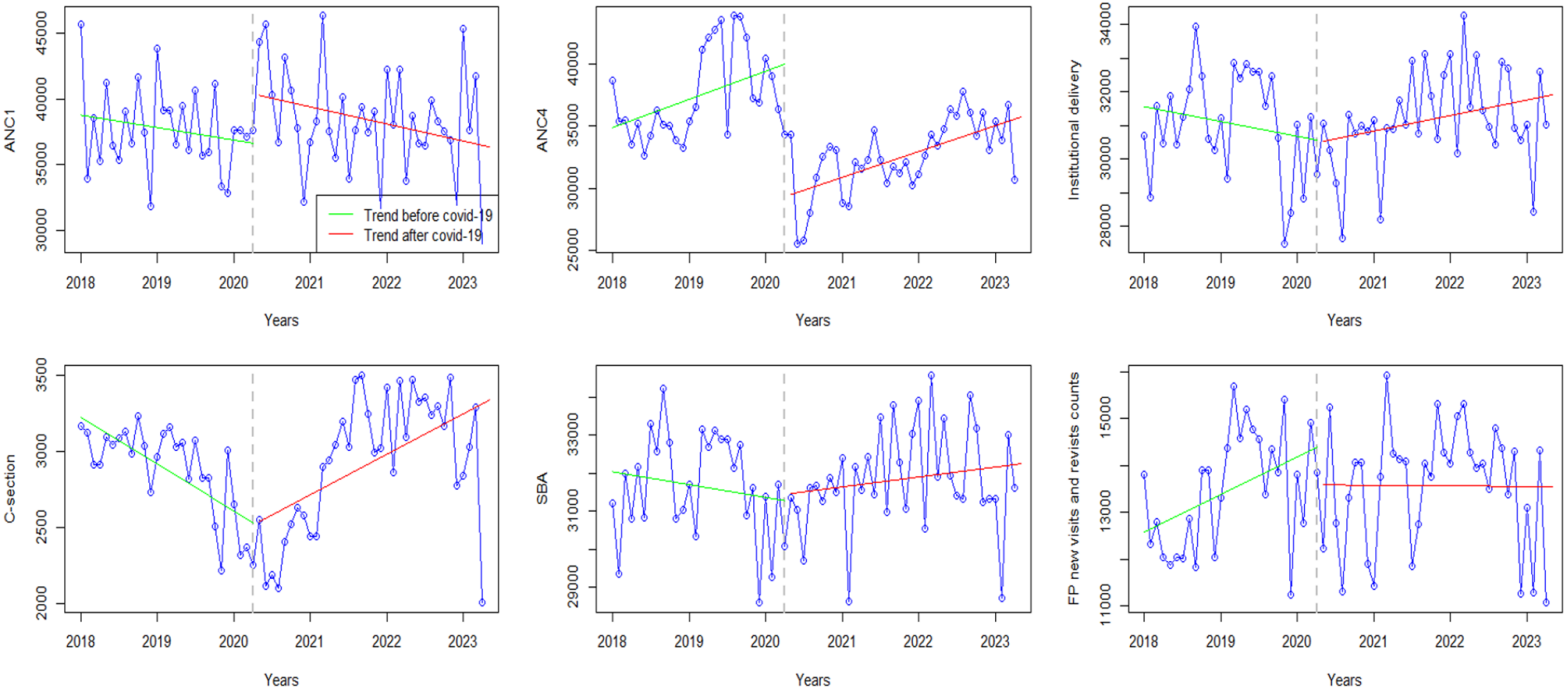
Visualization for maternal health pre versus post covid-19

Figure 1 shows the trends of MH indicators in Zimbabwe. Generally, the visualizations suggest a negative trend for most of the MH indicators except for the Caesarean section where a positive change in level was observed post-COVID-19. Before the pandemic, ANC visits were as high as above 40,000 but plunged to well below 35,000 both for the first and the fourth visits. Institutional deliveries decreased from 33,000 before COVID-19 to the lowest number of below 28,000 up to a maximum of around 31,000 during the pandemic. A similar trend was noted with skilled birth attendants (SBA). Before the pandemic, the number of new Family Planning visits was steadily increasing at an exponential rate. However, after the pandemic hit, there was a slight drop in the number of visits (from roughly 14100), and since then the number has remained fairly constant, hovering around 13,500 until 2023 (Fig. 1).

Similarly, the Key Informants respondents generally stated that the pandemic affected service delivery. For example, a nurse from a maternal and child health clinic at a general hospital had this to say about the issue:

*“Okay. There was some reduction in service delivery in terms of people being able to access the services. Because due to the*,* mostly due to the roadblocks and a bit of a friction between the local population and the enforcing law agents… so in terms of our department*,* most of the activities*,* they slowed down*,* we kind of shifted our focus more to COVID-19 activities…”****K10 Child and Maternal***.

### Immunization

**Fig. 2:**
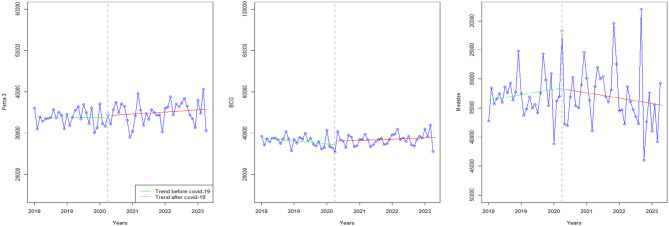
Visualization of immunization indicators pre versus post covid-19

Figure 2 displays the trends of Pentavalent 3 and Protection at Birth immunization in Zimbabwe. Both indicators seemed similar in level, before and after COVID-19’s onset in Zimbabwe; the trends remained stagnant after COVID-19’s onset (Fig. 2). However, measles immunization was inconsistent to Pentavalent 3 or BCG, showing a declining trend during the COVID-19 pandemic which persisted to post pandemic.

Key informants echoed similar sentiments generally highlighting declines in immunization activities. One of them had this to say:

*“We’re just following the guidelines for prevention of COVID when they are visiting the health facility. At a health facility*,* especially in rural areas*,* they were told*,* in some of the areas*,* they were told to say*,* village A*,* you come to the clinic on such a day*,* so that they will not just go congestion at the facility. Village B*,* you come to the health facilities on such and such a day within a catchment area. So*,* the nurse would map a catchment area and then identify and also tell his or her village health workers to date for vaccination*,* to reduce congestion at the health facility…”****– K2 EPI Manager.***

### Tuberculosis

**Fig. 3:**
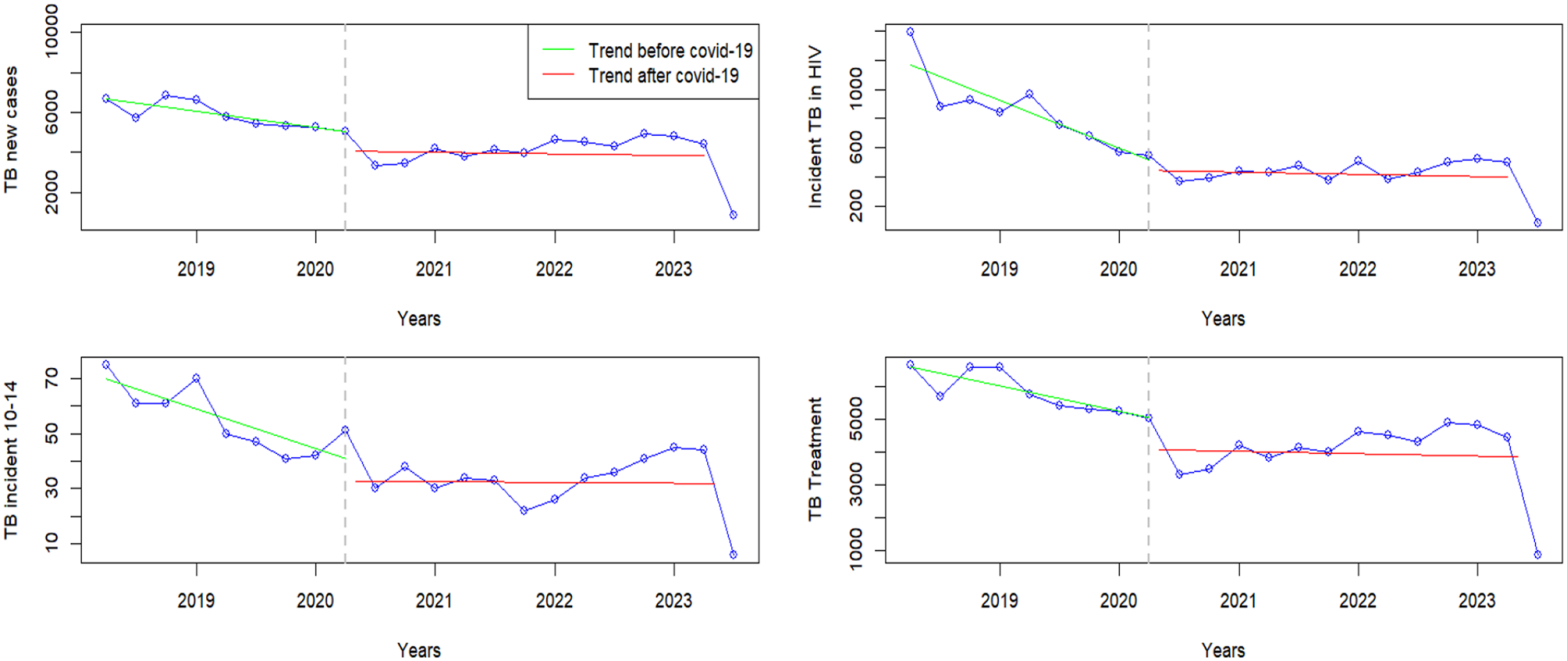
Visualization of TB indicators pre versus post covid-19

Figure 3 shows the trend before (green colour) and after (red colour) covid-19 of from right to left: New cases of TB, Incidents of TB, Incidents of TB among 0–14 years old, and the frequency of TB-infected individuals receiving treatment.

Compared to the period before the COVID-19 pandemic, which showed a decline in TB incidence, there was stagnation in TB incidence during and after the pandemic (even among the 10–14 age group, and incident TB in HIV), possibly due to limited access to TB services. This is further reflected in the graphs, which show a decline in patients accessing TB treatment from just above 5000 before COVID-19 to as low as 3000 (Fig. 3).

Key informant sentiments indicated similar disruptions to healthcare services, including access to essential medications like antiretrovirals (ARVs), during the COVID-19 pandemic, necessitating innovative approaches such as virtual meetings and community radio broadcasts to continue vital health education and support as expressed by one of them:

*“Even in terms of people accessing ARV it affected because some could not come or travel to get their ARV’s*,* so most people could not get the services they required… in terms of institution*,* there were a lot of modalities that were done*,* in terms of sensitized communities*,* we resorted to using our virtual zoom meetings*,* we resorted to using community radios*,* because as the national aids council we have contracts with almost every community radio station*,* so we are using those platforms now to sensitize communities about covid19 and any other health-related things…”****K13 TB.***

### Maternal and child mortality

**Fig. 4:**
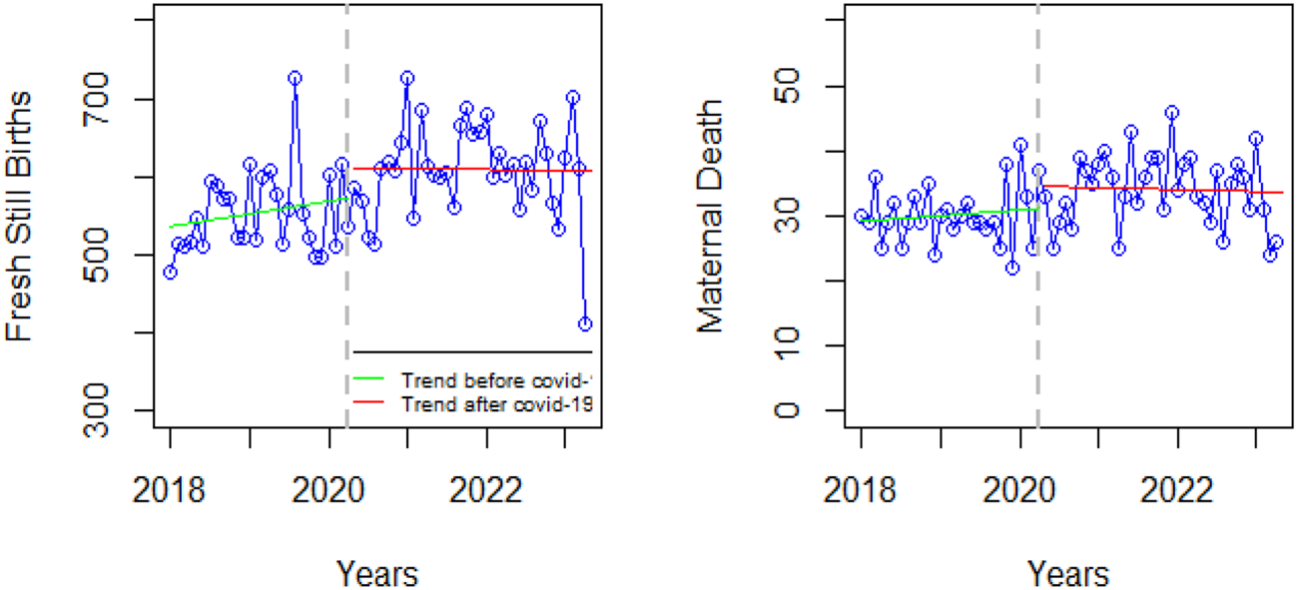
Visualization of mortality indicators pre versus post covid-19

There was a significant increase in level change in the number of maternal deaths. However, there was no significant slope change before and during COVID-19. Similarly, there was no difference in fresh stillbirths before and after the onset of COVID-19 (Fig. 4).

### Nutrition

**Fig. 5:**
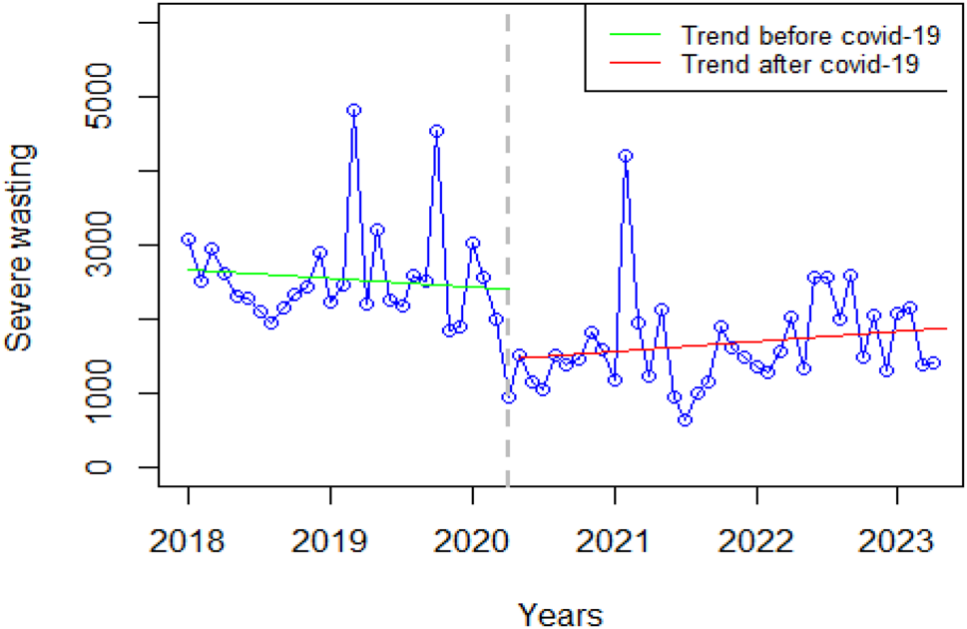
Visualization of nutrition indicators pre versus post covid-19

There was a significant decrease in severe wasting (WHO weight for height z-score < -3 for under five children) at the onset of COVID-19 and a slightly significant slope change for the pre-and COVID-19 periods (Fig. 5).

The key informants highlighted those restrictions led to limited healthcare-seeking behavior, posing challenges for individuals with non-life-threatening conditions like diarrhoea, exacerbating access barriers to essential medical services. They mentioned:

*“During the pandemic*,* there was limited movement of people. When someone has a condition that they felt was not life-threatening*,* they choose not to go to a facility. So*,* for example*,* when we said lockdown*,* it was a total lockdown*,* what happened to a child with probable diarrhoea*,* who was about to go to a facility that very day? It became difficult.”****K1 Nutrition***.

### Mental health

**Fig. 6:**
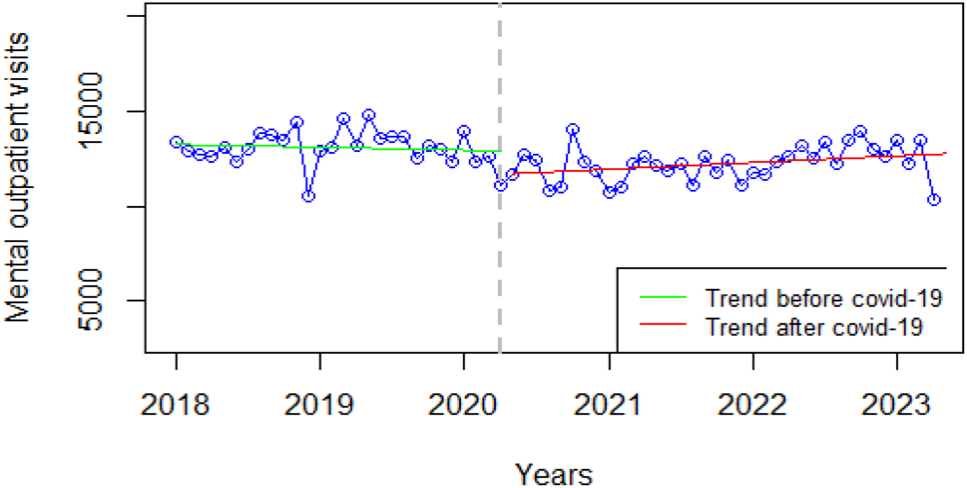
Visualization of mental health indicators pre versus post covid-19

There was a significant decrease in mental health outpatient visits at the onset of COVID-19; the same trend was maintained before and during the COVID-19 period (Fig. 6). Figure 6 shows a slight variation in the pre and post-COVID-19 outpatient visits for mental health services. However, the inclination is towards a slight decline in the number of mental health visits during the COVID-19 as compared to the post-COVID-19 period possibly indicating barriers to accessing care during the COVID-19 pandemic.

The key informants expressed a rather unclear picture of the issue of seeking outpatient mental health services as spelt out by one of them:

*“So*,* we had so many challenges with the permission to enter into certain provinces and certain districts. There was a lot of fear because there was no clear information. Notably*,* there were so many speculations more than what we could comprehend…. in the department*,* we are also spiritually inclined to certain beliefs. We had our fears about it and we didn’t know what could be the right position on sanitizers*,* and masks as these always changed”****K3 Mental Health***.

### Malaria

**Fig. 7:**
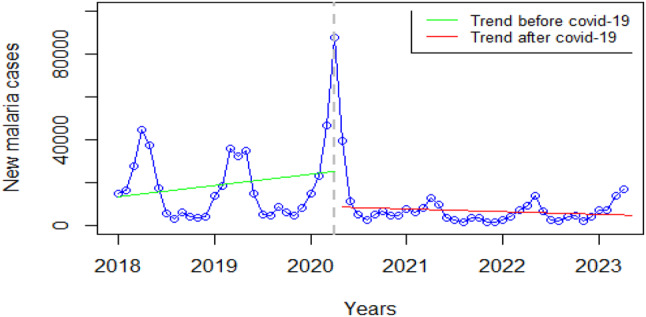
Visualization of malaria indicators pre versus post covid-19

There was a decrease in new malaria cases at the onset of COVID-19, followed by a sharp increase in the initial months of the COVID-19 pandemic from less than1000 to 8000 cases then the cases dropped within six months and the same low trend was maintained until after the acute COVID-19 period (Fig. 7).

Interviews with Key informants confirmed the trends as commented here:

*“At the beginning of the pandemic*,* we had challenges; some health workers were turning away our malaria patients away. So that had a negative impact. As a result*,* we saw our malaria cases nearly double and our incidence going up…. Also*,* the other issue that made cases increase a lot was a lot of travel restrictions that were imposed on the community such that someone might be sick and they’re not able to travel because of a lack of transport and health workers. Besides*,* the community health workers were running out of commodities*,* and they could also not travel”***K12 IEC Malaria.**

### Responses to COVID-19 pandemic

The respondents mentioned various mitigation strategies that were carried out in response to the pandemic. Some strategies include community-based services, outreach activities, training and capacity building, decongesting public places, and including all stakeholders. In addition to these mitigation strategies, the government followed the COVID-19 provision guidelines. Respondents echoed the following sentiments:

*“The efforts that were made*,* I think even for us as a National Aids Council*,* we also tried to ensure that the community partners who were out there in the community would become functional. They would be supplied with the prevention measures*,* and then they were really taught about COVID*,* and they would then visit and identify people who needed to go to the hospital and things like that…”-****K14 HIV.***

*“So*,* you see that the approach that was taken included almost every citizen and each leadership. And the involvement and participation of citizens and structures was wholesome…I think*,* like I said*,* that everything was COVID. We zeroed on to COVID*,* trained our staff on how to manage*,* ensured that all vacant posts were filled in*,* and there was also a COVID fund that was for resources…”****K6 Environmental Health***.

*“We’re just following the guidelines for prevention of COVID when they are visiting the health facility. And at a health facility*,* especially in rural areas*,* they were told*,* some of the areas*,* they were told to say*,* village A*,* you come to the clinic on such a day*,* so that they will not just go and cause congestion at the facility. Village B*,* you come to the health facilities on such and such a day within a catchment area. So*,* the nurse would map a catchment area and then identify and also tell his or her village health workers the date for vaccination*,* to reduce congestion at the health facility…”****– K2 EPI Manager.***

The different health departments also made modifications and played a role in responding to the COVID-19 pandemic.

“*Okay*,* so we developed a package which was called minimum initial service package whereby we are trying to get our different sectors under the SRH activities whereby we come up with a plan on what to do if we have a pandemic…”****K10 Child Maternal***.

*“I think in terms of institution*,* there were a lot of modalities that were done; in terms of sensitized communities*,* we resorted to using our virtual Zoom meetings*,* we resorted to using community radios*,* because as the national aids council*,* we have contracts with almost every community radio station*,* so we are using those platforms now to sensitize communities about covid19 and any other health-related things…”****K13 T***.

## Discussion

The results show that the COVID-19 pandemic negatively impacted delivery and access to healthcare services in part due to the restricted movement of clients and healthcare workers. Murewanhema and Makurumidze (2020) observed similar findings about disruption of healthcare services; delays in reaching out for health services by patients, disruption of critical health services such as maternal and child health and reproductive health issues, and other specialist services [27]. Similarly, in Ethiopia health facilities were repurposed and health care workers reshuffled [7]. The sudden drop in services observed in maternal and child health, immunization, tuberculosis, nutrition, mental health, and malaria is synonymous with trends observed globally [28–30]. However, some services like Caesarean-sections were observed to be on the rise from the national database.

Nonetheless, in support of the above findings, the World Health Organisation (2021) reiterated that preventive, curative services and supply chain management were negatively impacted by the pandemic. Disruption of services was also observed in the provision of mental health services where patients failed to access medicines and facilities due to travel restrictions [31]. In addition, it was observed that health personnel experienced burnout and were not capacitated to effectively provide services to patients. However, in the UK, mental health services uptake spiked during COVID-19, which bucked the trend of other services [29]. The rise in maternal and child mortality cases was in synchrony with the decline in service delivery during the pandemic. The same trend was observed in several low and middle-income countries [32, 33]. However, other services like Pentavalent 3 or BCG immunization remained unchanged after COVID-19 pandemic may be due to their provision at birth/ within 28 days, making them a priority in routine healthcare services to prevent vaccine-preventable diseases such that there was a lower chance of the vaccinations being missed.

The study indicated several mitigation strategies that were introduced during the pandemic, and these included the use of community-based services, outreach services, capacity building, and de-congestion of public services. Mitigation strategies were not only peculiar to the Zimbabwean context. In Ethiopia, the US, and the UK, most services recovered as systems were put in place including capacity development of the health workforce, improvement of conducive working conditions, provision of PPE, and building resilience within the health workforce through social dialogue [7, 34, 35]. A key limitation of the study is that it did not recruit community representatives or local people who could have provided an important perspective on access to health services during the COVID-19 pandemic as they were not part of the inclusion criteria (healthcare workers). In addition, the choice of indicators was not exhaustive, as it covered only a selected health services, potentially overlooking other relevant factors that could have provided a more comprehensive understanding of the impact of the COVID-19 pandemic.

## Conclusions

The COVID-19 pandemic had a negative impact on health service delivery. Disruptions of critical healthcare services, maternal and child health, reproductive health issues, and other specialist services were prominent. In addition, health personnel experienced burnout and were not sufficiently capacitated to effectively provide services to clients within the pandemic environment. The rise in maternal and child mortality cases and caesarean sections could be directly linked to the decline in service delivery during the pandemic. Mitigation strategies that were introduced during the pandemic included the use of community-based services, outreach services, capacity building, and de-congestion of public services.

### Recommendations

The study would recommend mitigation strategies that were introduced during the pandemic period including (i) the use of community-based services (ii) outreach services (iii) de-congestion of public services (iv) capacity development and building resilience within the health workforce in preparation for future pandemics and unforeseen health emergencies: improvement of conducive working conditions, provision of PPE and preparatory continuing workshop for future emergencies.

## Electronic supplementary material

Below is the link to the electronic supplementary material.

Supplementary Material 1

## Data Availability

The data that support the findings of this study are housed at the Infectious Diseases Institute, Makerere University and available on reasonable request from the corresponding author. The data are not publicly available due to privacy/ethical restrictions.

## Abbreviations

AIC: Akaike Information Criterion
ACF: Autocorrelation Function
ARV: Antiretrovirals
BCG: Bacillus Calmette-Guérin
COVID-19: Coronavirus Disease
DHIS2: District Health Information Systems 2
HIV: Human Immunodeficiency Virus
KII: Key Informant Interviews
MH: Maternal Health
MoHCC: Ministry of Health and Child Care
PACF: Partial Autocorrelation Function
SBA: Skilled Birth Attendants
SARS-CoV-2: Severe Acute Respiratory Syndrome Coronavirus 2
TB: Tuberculosis
WHO: World Health Organization
