# Supplementary material for: Impact of COVID-19 on healthcare programs in Zimbabwe: a mixed methods study
Source: BMC Public Health. 2025 May 13;25:1749. doi: 10.1186/s12889-025-22791-4 (PMC12070714; doi:10.1186/s12889-025-22791-4)
Supplement: Supplementary file 1 — Supplementary Material 1 [file 12889_2025_22791_MOESM1_ESM.pdf]

MINISTRY OF HEALTH AND CHILD CARE
NATIONAL INSTITUTE OF HEALTH RESEARCH
Josiah Tongogara Avenue/Mazowe Street
P O Box CY 573
Causeway
Harare

MRCZ/A/2987

Program on COVID-19 Vaccine Effectiveness, Vaccine Uptake Determinants, and Impact of Pandemic on Health Programs in Zimbabwe

KEY INFORMANT INTERVIEW GUIDE (English version)

MEDICAL RESEARCH COUNCIL OF ZIMBABWE
APPROVED
16 FEB 2023
20 CAMBRIDGE ROAD
AVONDALE, HARARE

Background and Informed Consent

Dear Colleague,

We are reaching out to you to assess how COVID-19 affected the different essential health services in Zimbabwe. To meet the goals of the African Union's vaccine strategy and to address the challenges facing the African continent, Africa CDC and Master Card Foundation launched the Saving Lives and Livelihoods (SLL) Programme in June 2021. The guiding principles of the programme include inclusivity of all African countries, equity reflecting countries' needs, optimal accountability, African-led through empowerment of AU member states, multi-partner collaboration, improvements to national health systems and application of a test and learn approach.

Ministry of Health and Child Care, University of Zimbabwe are partners of SLL under the implementation science pillar, implementing the Program for Research on Vaccine Effectiveness (PROVE). This program is mandated to build the capacity of the National Institutes of Public Health (NIPHs) of African Union (AU) member states to combat pandemics. PROVE aims to assess; the effectiveness of COVID19 vaccination, barriers, and enablers to COVID19 vaccination, and the impact of COVID19 on the health programs in Africa.

Our aim for this interview is to assess the impact of the COVID-19 pandemic on the health systems in Zimbabwe, focusing on key thematic areas including maternal and child health services, HIV/TB, blood donation services, Malaria, and routine immunization programs, among others. This information will help the decision makers understand the common issues to be addressed in our health system and develop strategies to strengthen the Zimbabwean health system to combat pandemics effectively.

Your responses from this interview will be kept confidential, and the information you give us will not jeopardize your work. We greatly appreciate your time and effort in responding to these questions. We may reach out to you again for further clarifications. Please confirm your consent for participation in this interview.

- ○ I agree to participate
- ○ I do not agree to participate

General information

Country	
Institution	
Position/designation	
Telephone contact	

Interview guide

**Section A: Socio-demographic data**	
1. Please introduce yourself.<br>2. What section of the health system do you support?<br>3. Gender of the participant:<br>4. Section of essential services being represented	
6. Date of Interview:	☐☐ ☐☐ ☐☐☐☐<br>Date Month Year
10. Initials of Interviewer:	....

Section B: Guiding questions related to COVID-19 and health services utilization during the COVID-19 pandemic.

- How has the situation of COVID-19 evolved in Zimbabwe? Please explain in the context of Zimbabwe.
- Are there any COVID-19 cases in your community? Please estimate the number of cases

- What was/is the provision of receiving COVID-19-related health services in your community? Please explain.
- In your observation or experience, how did people in Zimbabwe utilize routine health services during the thick of the COVID-19 pandemic?
- Could you please share your experiences or efforts in ensuring the continued delivery of the section of the health system you support despite the pandemic?
- In your experiences or observation, what were the effects on the general health services availability before, during and after the lockdown period? Can you please also provide some examples?
- How did vulnerable people, including mothers, children, and the elderly, continue to receive health services during this pandemic in Zimbabwe?
    - -Probing to explore more with some examples.
- In your observation or experiences, how are the different levels of the government responding to this pandemic, particularly in providing health services to community people?
- How do you think COVID-19 affected service delivery at your department, institutional and country levels?
- What health service areas should be prioritized for future pandemic control?
- What modifications were made in health care planning and financing within your institution/department to inform pandemic control and management?
- What challenges did you encounter during the pandemic that have informed your insights on health service delivery for the future?
- In your view, what strategic decisions and systems need to be implemented to guard against pandemics?
- Please feel free to share if you have any suggestions or anything you would like to say or think you have missed during our conversation.

Closing remarks

Thank you for your participation, good-bye.

MEDICAL RESEARCH COUNCIL OF ZIMBABWE
APPROVED
16 FEB 2023
20 CAMBRIDGE ROAD
AVONDALE, HARARE
